# Comparative analysis of genomic- and EST-SSRs in European plum (*Prunus domestica* L.): implications for the diversity analysis of polyploids

**DOI:** 10.1007/s13205-020-02513-w

**Published:** 2020-11-21

**Authors:** Rosanna Manco, Pasquale Chiaiese, Boris Basile, Giandomenico Corrado

**Affiliations:** Dipartimento di Agraria, Università Degli Studi di Napoli Federico II, via Università 100, 80055 Portici, NA Italy

**Keywords:** Stone fruit, Polyploidy, Polymorphism, Diversity, Microsatellites

## Abstract

Simple sequence repeats (SSRs) are among the most useful DNA markers in plant science. The aim of this study was to compare the features and usefulness of genomic SSR (gSSR) and EST-SSRs in European plum (*Prunus domestica* L.), an economically important, hexaploid stone fruit crop globally cultivated to produce fleshy fruits and derived foodstuff. The analysis of an ample set of morphologically diverse varieties indicated that gSSRs and EST-SSRs provide different estimates of some of the locus-based indicators of diversity. Moreover, the two classes of SSRs gave different, weakly correlated, estimations of distance-based parameters with gSSRs being more powerful for discriminating purposes. The two SSR classes provide complementary information in European plum, making the contribution of EST-SSRs useful not only as non-neutral markers. The differences between SSR classes are discussed considering the neutral and non-neutral evolution, and the polyploidy and asexual propagation of the cultivated tree varieties.

**Electronic supplementary material:**

The online version of this article (10.1007/s13205-020-02513-w) contains supplementary material, which is available to authorized users.

## Introduction

European plum (*Prunus domestica* L.) is an economically important stone fruit crop, globally cultivated in temperate areas for its fleshy fruits. These are mainly marketed fresh, canned or dried (Neumüller 2011). Further uses include the production of juices, fruit brandy, and flavours for jams, candies, sweets, and other baked foods. Commercially available European plum varieties present a range of phenotypic traits (e.g., fruits shape and size; skin and flesh colour; firmness; taste) that originated several classifications. Cultivated varieties are typically classified in different pomological groups by breeders and retailers, although the degree of overlap of morphological traits and the complex interspecific origin of the species do not always allow a clear distinction among all the different groups (Zhebentyayeva et al. 2019).

Considerable progress in describing and classifying the ample diversity of the European plum has been achieved with the introduction of DNA molecular markers (Decroocq et al. 2004; Dirlewanger et al. 2002; Li et al. 2010; Shimada et al. 1999). These analyses also confirmed that the European plum clade of *P. domestica* has a high level of diversity (Zhebentyayeva et al. 2019). Moreover, DNA markers have been used in plum for different purposes, such as map-based cloning (Claverie et al. 2004), population structure analysis (Horvath et al. 2011), phylogenetic relationships (Liu et al. 2007; Reales et al. 2010), landraces examination (Manco et al. 2019; Sehic et al. 2015), and discrimination of clones (Gharbi et al. 2014).

Microsatellites, also known in plant genetics as simple sequence repeats (SSRs), are one of the most suitable markers for *Prunus* diversity (Aranzana et al. 2019), being multiallelic and highly polymorphic. The diffusion of the SSRs in genetics is also due to their good transferability across similar species (Mnejja et al. 2010). SSRs are DNA sequences with a simple core motif of one to six nucleotides that is tandemly repeated. Microsatellites are frequently used for orphan plants (e.g., those without a publicly available reference genome sequence) and for polyploid and highly heterozygous species because of their codominant nature. Considering that SSRs have been traditionally isolated from genomic libraries enriched in repetitive sequences, they are usually treated as neutral markers (Goldstein and Schlötterer 1999; Ellegren 2004). For these reasons, SSRs are highly informative to investigate clonality, identify genotypes, and describe demographic process such as genetic drift and migration (Goldstein and Schlötterer 1999; Ellegren 2004).

Recent technical advances in genomics along with the improvement of computational statistical tools allow the evaluation of nucleotide polymorphisms at the genome scale, thus overcoming some of the limitations of the SSR analysis (e.g., cost of the SSR development, low-medium throughput, and risk of technical artefacts). High-throughput sequencing (HTS) is currently changing SSR profiling in human forensics (Børsting and Morling 2015; Parson et al. 2016). In spite of the problems of sequencing highly repetitive long DNA sequences (Treangen and Salzberg 2012), a key advancement of the HTS is the ability to directly reveal a high number of (short) microsatellites sequences with a single assay (Šarhanová et al. 2018). Moreover, HTS and more specifically, the RNA-Seq is a much more affordable approach to identify SSRs at a fraction of the cost of the traditional cloning-based strategy or the whole genome sequencing and assembly (Taheri et al. 2018; Martin et al. 2013). The number of the EST-SSRs (i.e. SSR markers part of a transcribed DNA sequence) identified from RNA-Seq efforts in orphan species has greatly increased, rendering less needed the use of transferable SSRs (Hodel et al. 2016a). EST-SSRs are therefore treated as non-neutral markers, useful for instance, to study adaptive genetic diversity (Ellis and Burke 2007). It is expected that the reduction of the Next Generation Sequencing (NGS) cost will increase the diffusion of this approach for non-model tree crops. This will allow to uncover DNA polymorphisms at an unprecedented scale by making available large data on both genomic (gSSRs) and EST-SSRs.

The present study was undertaken to evaluate the differences between the features and the information provided by different classes of SSRs in cultivated European plum. *P. domestica* is polyploid (2n=6x=46) and agamically propagated in agriculture. These features can significantly impact the evaluation of various genetic parameters (Meirmans et al. 2018). In order to provide information useful for the analysis of genetic diversity and for association studies in *P. domestica*, we investigated the differences between EST-SSRs and gSSRs in revealing genetic diversity considering a common large set of European plum varieties.

## Materials and methods

### DNA analysis

The DNA analysis was carried out from young leaves of 44 European plum (*Prunus domestica* L.) Italian varieties namely: ‘Biancolella di Ottaviano’ (Bian), ‘Botta a muro bianca’ (Bott), ‘Cacazzara’ (Caca), ‘Calavrice’ (Cala), ‘Coglie e astag bianca’ (CogB), ‘Coglie e astag nera’ (Cogn), ‘Core’ (Core), ‘Del Carmine’ (Delc), ‘Della Maddalena’ (Dell), ‘Di Spagna’ (Diso), ‘Fele’ (Fele), ‘Fiaschetta’ (Fias), ‘Fiocco bianco’ (Fiob), ‘Fiocco rosa’ (Fioc), ‘Genova giallo-verde’ (Geno), ‘Lecina tonda’ (Leci), ‘Marchigiana’ (Marc), ‘Mbriaca’ (Mbri), ‘Melella’ (Mele), ‘Nera tardiva’ (Nera), ‘Occhio di bue’ (Occh), ‘Pannaranese’ (Pann), ‘Pappacona’ (Papp), ‘Pappacona gialla’ (Papg), ‘Pappacona rossa’ (Papr), ‘Pappacona verde’ (Papv), ‘Pazza di Somma’ (Pazz), ‘Pezza rossa’ (Pezz), ‘Preta ‘e zucchero’ (Pret), ‘Prunarinia’ (Prun), ‘Rachele’ (Rach), ‘Riardo’ (Riar), ‘San Giovanni’ (Sang), ‘San Rafele’ (Sanr), ‘Santa Maria’ (Sanm), ‘Santangiolese’ (Sana), ‘Santa Paola’ (Sanp), ‘Scarrafona’ (Scar), ‘Scauratella’ (Scau), ‘Sile’ (Sile), ‘Turcona’ (Turc), ‘Uttaiana’ (Utta), ‘Zi’ ‘Augusto’ (Ziau), ‘Zuccarina’ (Zucc). Main morphological features of the plums are reported in Supplementary Table 1. Samples were obtained from the field collection of the ‘Azienda Agricola Sperimentale Regionale Improsta’ of the ‘Centro per la Ricerca Applicata in Agricoltura’ (C.R.A.A.) of the Campania region of Italy. We analysed two different plants per variety. DNA isolation, quantification and amplification were performed as previously described (Manco et al. 2019).

The molecular fingerprint was carried out using five SSRs from EST-libraries (EST-SSRs) and five SSRs from genomic libraries (gSSRs) selected from different sources to avoid possible bias related to their identification and selection. The EST-SSRs loci were EPPISF001, EPPISF004, EPPISF027 (Vendramin et al. 2007), ES4 and ES5 (Li et al. 2010). The gSSRs loci were BPPCT 004, BPPCT 014, BPPCT 028 (Dirlewanger et al. 2002), PS12A02 (Downey and Iezzoni 2000), and UDP98-409 (Cipriani et al. 1999). For each publication, loci were selected based on the number of alleles detected in *P. domestica*. Primer sequences and annealing temperature are reported in Supplementary Table 2. PCR amplification and capillary electrophoresis were performed as described (Manco et al. 2019) using an ABI Prism 3130 Genetic Analyzer system (Applied Biosystems). Raw sizes were calculated with the local Southern algorithm implemented in the GeneMapper 4.0 software (Applied Biosystems) using the GeneScan 500 LIZ Size Standard (Applied Biosystems). Values were rounded to integer and scaled according to the SSR-core motif (Supplementary Table 2), while minimizing the average offset of the alleles for each SSR within the instrumental resolution (± 1 bp).

### Locus-based data analysis

For each SSR, we calculated the number of alleles; the Simpson's index of diversity (1-D), where D is (1-Σ*p*_i_), with *p*_i_ being the proportion of the i-th allele of a locus; and the evenness (Ev), calculated as: [(1/λ)-1]/[(e^H)-1], where 1/λ is Stoddart and Taylor’s index and H is the Shannon diversity. The genotype accumulation curve was created by randomly sampling (*n* = 10,000) loci to create the distribution, and then counting the number of multilocus genotypes (MLG) for an increasing number of SSRs (from 1 to n–1, where n is the maximum number of markers). These calculations were performed using the poppr library in the R environment (Kamvar et al. 2014).

The effective number of alleles (En; the number of alleles in a population weighed for their frequencies), the gametic heterozygosity (GH; the chance that two random alleles drawn from the individual are the same) (Moody et al. 1993), and the expected heterozygosity (Hs; the expected frequency of heterozygotes within a population) were calculated with the GenoDive software (Meirmans and Van Tienderen 2004).

The allelic richness (AR), expressed as the expected number of alleles among *k* gene copies (*k* = 79 EST-SSRs; *k* = 82 gSSRs), the gene diversity (GD) corrected for sample size, and the observed heterozygosity (Ho) were calculated with SPAGeDi v. 1.5 (Hardy and Vekemans 2002). For each locus, we also statistically evaluate the ratio (R) between the effective number of alleles and the total number of alleles using the *z* test statistics based on a random generation for the normal distribution (pnorm) implemented in R (Team R Core 2013).

### Distance-based data analysis

Pairwise genetic distances were calculated with the Bruvo’s coefficient, because this index was developed to analyse microsatellite data from polyploids (Bruvo et al. 2004), utilizing the combinational model, implemented in the poppr library (Kamvar et al. 2014). The agglomerative hierarchical clustering was performed by applying the unweighted pair-group method with arithmetic averages (UPGMA) algorithm implemented in R using the Bruvo’s distances. To statistically test the linear correlation between the molecular distances, the two parallel matrices from gSSR and EST-SSRs were compared by a Mantel test with 9999 permutations. Tree topologies were compared considering the parallel matrices with cophenetic values. A tanglegram plot of a side by side tree representation was created with the dendextend library in the R environment (Galili 2015).

## Results

### Differences in locus-based parameters

The DNA fingerprint of the 44 cultivated varieties indicated that all the EST-SSRs were polymorphic in our germplasm collection. Main genetic parameters are presented in Table 1. The EST-derived SSRs generated on average 9.60 alleles per locus, ranging from a minimum of six (ES4) to a maximum of 12 (EPPISF001). The Simpson’s index of diversity (1–D) was high and displayed little variation across loci (CV = 5.74%), while a higher variation across loci was present for the evenness (CV = 15.6%), a parameter that measures the distribution of genotype abundance in a population. The number of alleles was positively correlated to the evenness (*r* = 0.717, *p* < 0.05; Pearson correlation coefficient) yet, the locus that distributed the alleles most uniformly in the varieties under investigation was the one with the least alleles (ES4). Considering that in a hexaploidy species the overall level of heterozygosity is not frequently due to full heterozygote loci, we calculated also the gametic heterozygosity (GD), a parameter that is weighted considering the different partial heterozygotes. As expected, the observed homozygosity was close to 1.0 for many loci, while the GD was lower and little differed across loci (CV = 8.3%). To account for the ploidy when estimating genetic diversity, the expected heterozygosity (Hs) was calculated with correction for missing dosage. This index was on average high (0.74) and did not correlate with the number of alleles in each locus.

**Table 1:** Main genetic indices for the EST-SSRs (locus) and their average values (mean)

Locus	An	1-D	Ev	AR	GD	Ho	GH	Hs	En	*R*
EPPISF001	12	0.82	0.73	11.07	0.79	0.99	0.66	0.81	5.15	0.43
EPPISF004	11	0.73	0.62	10.24	0.67	1.00	0.66	0.58	2.36	0.21**
EPPISF027	8	0.70	0.71	7.67	0.66	1.00	0.65	0.65	2.77	0.35*
ES4	6	0.75	0.83	6.00	0.72	0.98	0.47	0.69	3.14	0.52
ES5	11	0.71	0.55	10.88	0.65	0.78	1.00	0.97	2.90	0.26**
Mean	9.6	0.74	0.69	9.17	0.70	0.95	0.69	0.74	3.26	0.36

*An* number of alleles; 1-D: Simpson’s index of diversity; *Ev* genotypic evenness; *AR* allelic richness; *GD* gene diversity; *Ho* observed heterozygosity: *GH* gametic heterozygosity; *Hs* expected heterozygosity; *En* effective number of allele; *R: En/An* asterisks indicate a statistically significant deviation relative to a normal distribution (*p < 0.05; **p < 0.01)

The gSSR were all polymorphic. The number of alleles of the gSSR (*n* = 96) was significantly higher (*p* < 0.001; *t* test) and overall, double than the number of EST-SSRs (*n* = 48), but with a similar CV across loci (Table 2). The number of gSSR alleles ranged from a minimum of 14, a value higher than the maximum number of alleles for EST-SSRs. Likewise, also allelic richness, the Simpson’s index of diversity, and the gene diversity were significantly higher for gSSRs (*p* < 0.001; *t* test), indicating that the genetic diversity captured by the gSSRs is higher. On the other hand, the evenness did not display a significant difference between the two kinds of SSRs (*p* > 0.05; *t* test). The observed heterozygosity (Ho) was for many loci close to the maximum value, similarly to the EST-SSRs, and also the gametic heterozygosity did not differ between the two SSR classes (*p* > 0.05; *t* test). These data suggest that differences in the number of alleles did not strongly influence their distribution in the analysed population.

**Table 2:** Main genetic indices for the gSSRs (locus) and their average values (mean)

Locus	An	1-D	Ev	AR	GD	Ho	GH	Hs	En	*R*
BPPCT004	17	0.90	0.80	15.49	0.89	1.00	0.88	0.88	8.25	0.49
BPPCT014	26	0.92	0.70	24.13	0.91	0.94	0.59	0.85	6.24	0.24**
BPPCT028	14	0.77	0.54	14.00	0.74	0.73	1.00	1.00	2.45	0.17**
PS12A02	21	0.92	0.80	19.27	0.91	1.00	0.72	0.91	10.42	0.47
UDP98409	18	0.89	0.76	16.72	0.88	1.00	0.59	0.85	6.30	0.35*
Mean	19.2	0.88	0.72	17.92	0.87	0.94	0.76	0.90	6.73	0.34

*An* number of alleles; *1-D* Simpson’s index of diversity; *Ev* genotypic evenness; *AR* allelic richness; *GD* gene diversity; *Ho* observed heterozygosity: *GH* gametic heterozygosity; *Hs* expected heterozygosity; *En* effective number of allele; R: *En/An* asterisks indicate a statistically significant deviation relative to a normal distribution (*p < 0.05; **p < 0.01)

The effective number of alleles (En) for both marker classes was substantially lower than the number of alleles, and did not significantly correlate with the observed number of alleles (*r* = 0,36; *p* > 0.05 for EST-SSRs; *r* = 0.42; *p* > 0.05 for gSSRs; Pearson correlation coefficient). However, in absolute terms, En differed between marker classes (*p* < 0.05; *t* test). To account for the different numbers of alleles between EST-SSRs and gSSRs, we evaluated the ratio (*R*) between En and An. As expected, there were locus-specific differences in both SSRs classes but the average R and the number of loci with a significantly low R did not differ between EST-SSRs and gSSRs.

**Fig. 1:**
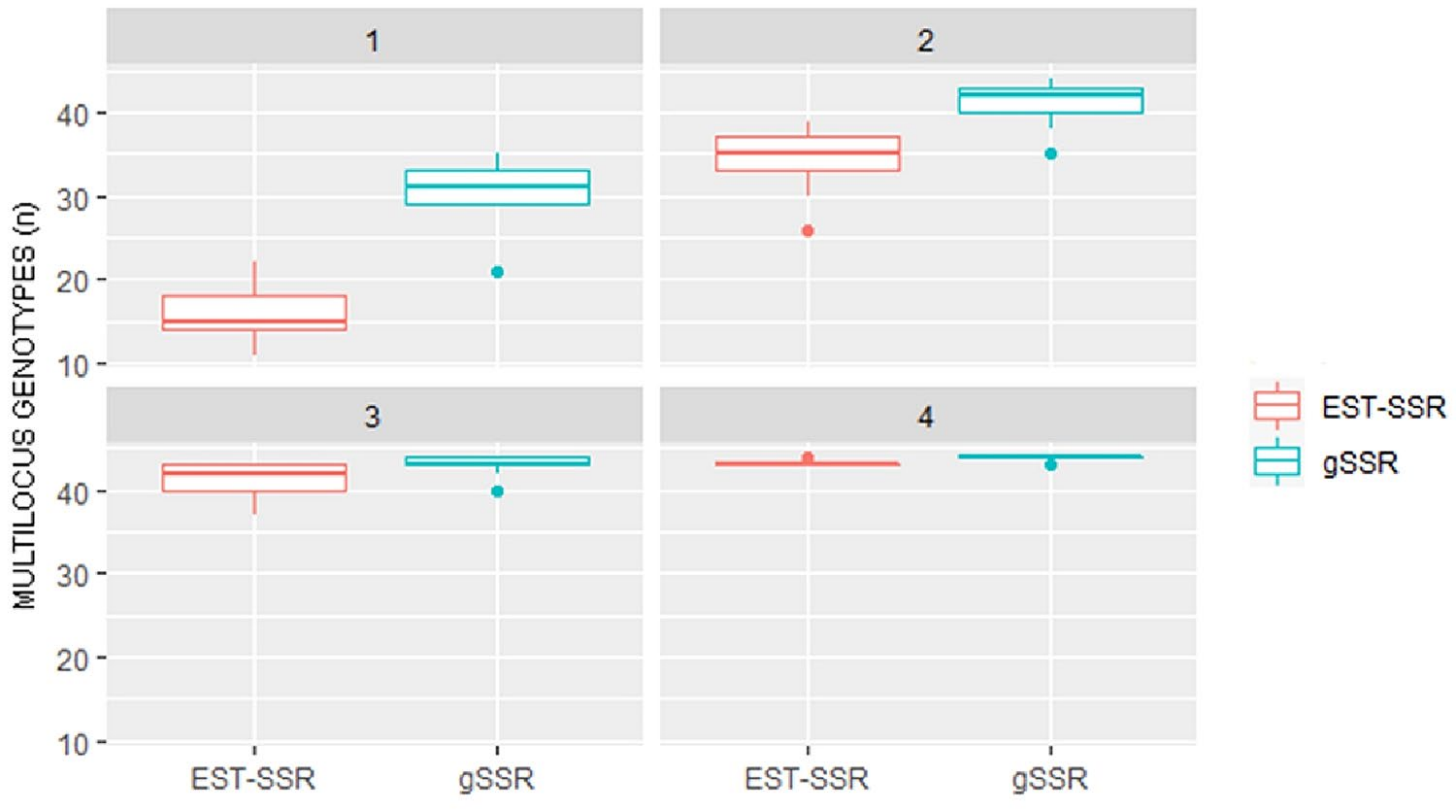
Genotype accumulation plot. The graph shows in different panels (representing increasing number of SSR loci as indicated in the top dark grey bar), a box-and-whisker plot of the number of multilocus genotypes obtained by randomly sampling loci without replacement (*n* = 10,000). The band inside the box represents the median (2nd quartile). Dots indicate outliers (i.e., values outside 1.5 times the interquartile range above the upper and below the lower quartile). EST-SSRs (resp., gSSRs) box-and-whisker plots are in salmon (resp., turquoise) colour

To analyse the ability of the SSRs in discriminating between unique genotypes according to their number, we performed an accumulation analysis (Fig. 1). The genotype accumulation for an increasing number of EST-SSRs or gSSR indicated that gSSR are always able to capture a higher number of multilocus genotypes (MLG). As expected, this difference gets smaller when reaching the plateau, i.e., the maximum number of MLG (44). The data indicated that gSSRs have a greater discriminatory power in European plum but also that, for the EST-SSRs, the addition of a small number of markers can yield a similar genotyping ability.

### Differences in distance-based parameters

**Fig. 2:**
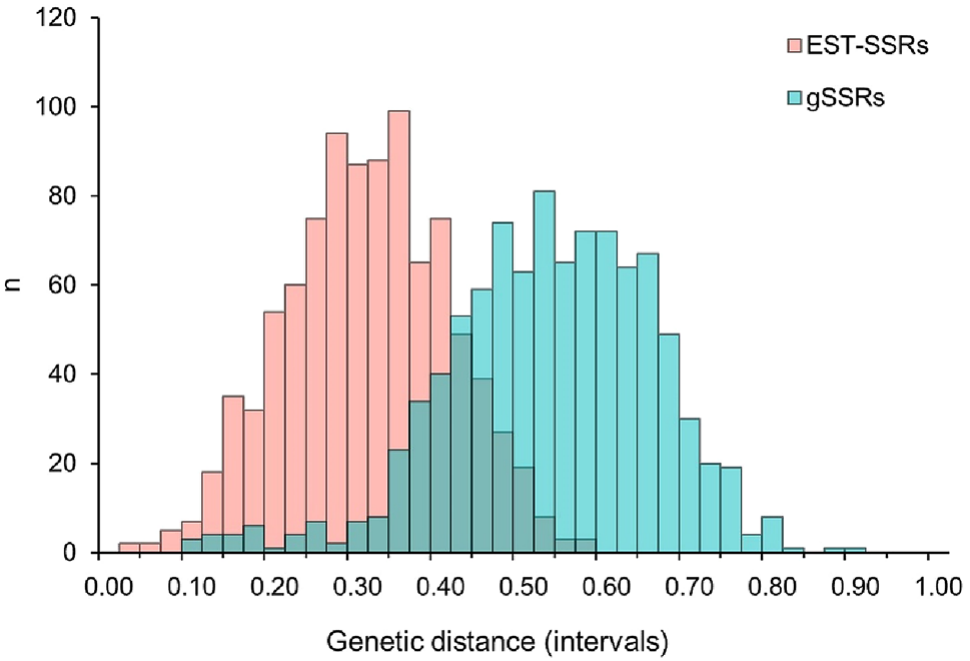
Distribution of the pairwise genetic distance among varieties calculated with EST-SSRs (salmon) or gSSRs (turquoise). Each bar shows the number of pairwise comparisons between the 44 varieties that have a genetic distance (calculated with the Bruvo’s index) included in the class intervals reported on the x-axis

**Fig. 3:**
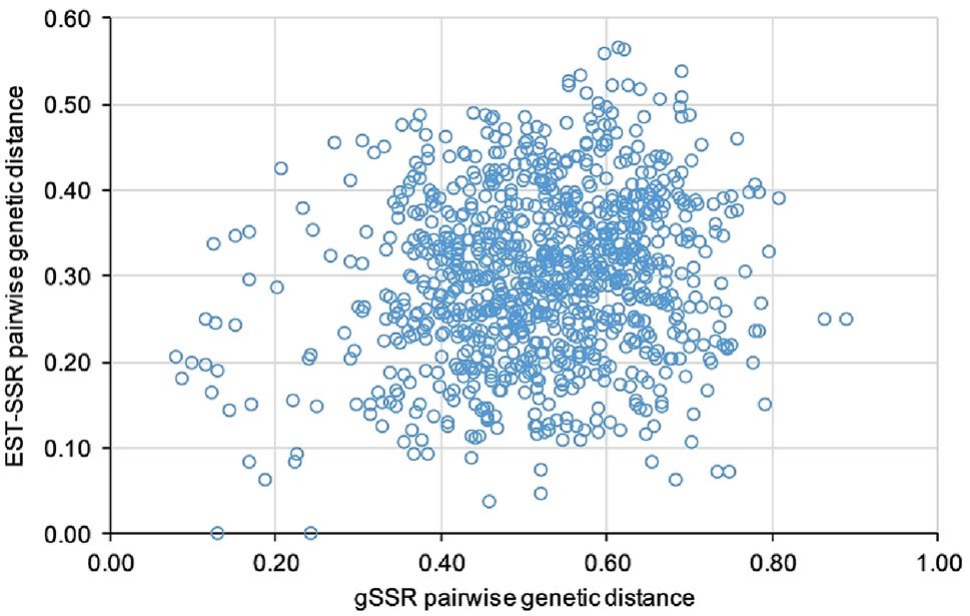
Scatterplot displaying the correlation between the genetic distances calculated from EST-SSRs (x-axis) and gSSRs (y-axis). The two coordinates of each circle are the genetic distances of a pairwise comparison between varieties

Due to their codominance, SSRs are highly valued to obtain matrices of genetic resemblance especially for highly heterozygotes genotypes, for instance, through the construction of dendrograms. To investigate the ability to describe the genetic differences among European plum genotypes, we calculated the Bruvo’s distances based on either EST-SSRs or gSSRs. The average Bruvo’s distance was higher for the gSSR (*p* < 0.001; *t* test) (Fig. 2). Moreover, gSSRs were able to discriminate all the varieties under investigation, while the EST-SSRs could not discriminate between two pairs of cultivars (‘Occhio di bue’ and ‘Fiocco bianco’; ‘Uttaiana e ‘Della Maddalena’). The distances based on gSSRs or EST-SSRs were both normally distributed (*p* > 0.05, Kolmogorov–Smirnov test of normality) (Supplementary Fig. 1). The correlation between the two distance matrices was low (*r* = 0.167) and statistically significant (*p* < 0.001; Mantel’s test). Moreover, a linear trend or a triangular shape of the scatterplot was not evident (Fig. 3). Lower pairwise distances obtained with one class of SSRs did not largely associate to lower pairwise distances with the other class of SSRs. Similarly, higher distances with one SRR class did not necessarily associate with higher distances obtained with the other thus, not giving rise to a triangular-shaped scatterplot expected when genotype pairs with low (respectively, high) level of diversity relative to one marker type show low (resp., high) level of diversity relative to another one. This implies that the higher distances provided by the gSSRs are not simply a linear extension of the genetic distance revealed by the EST-SSRs.

**Fig. 4:**
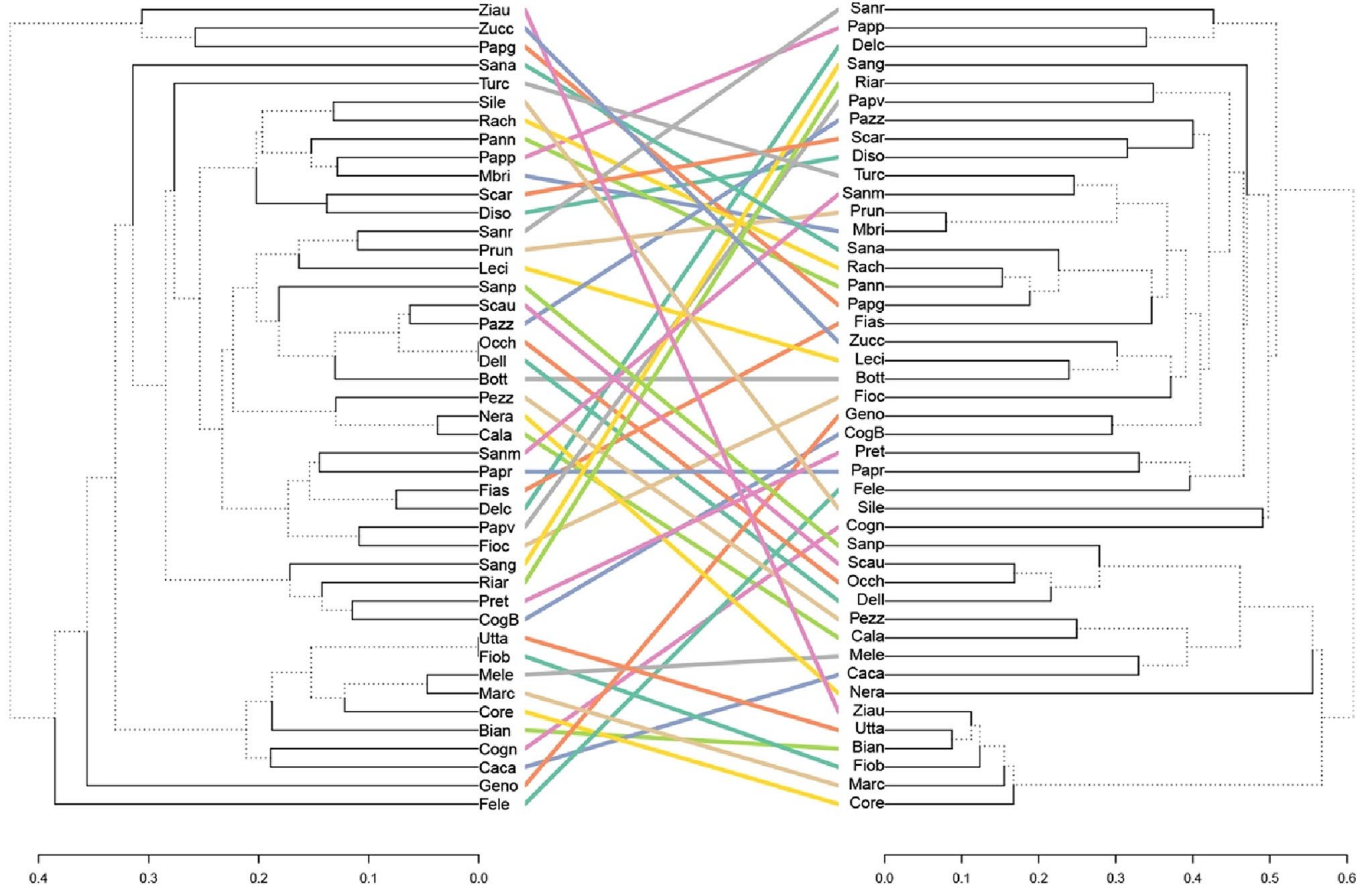
A comparison of the hierarchical cluster analysis (HCA) of the plum varieties using EST-SSRs (left) or gSSRs (right) data. HCA was performed with the UPGMA algorithm for both dendrograms, using the Bruvo’s genetic distances. To ease the comparison, coloured lines connect identical names. The different line type in the dendrograms highlights distinct edges in a tree (compared to the other one)

To illustrate possible differences in describing genetic diversity, we built and compared dendrograms from hierarchical clustering based on the Bruvo’s distance. The cophenetic correlation between the trees was positive (*r* = 0.143; *p* < 0.05, Mantel) and slightly lower than the correlation of the genetic distances (Fig. 4). The result indicated that in plum the genetic resemblance depicted with EST-SSRs did not largely relate with that obtained with gSSRs.

## Discussion

SSRs are one of the highly polymorphic and versatile DNA markers for plants (Hodel et al. 2016b; Vieira et al. 2016). Although their genome coverage remains lower than that of SNPs, the advent of NGS technologies largely expanded the number of available microsatellites, especially those identified in transcribed sequences. The usefulness of microsatellite in European plum has been previously demonstrated (Decroocq et al. 2004; Dirlewanger et al. 2002; Li et al. 2010). Considering that advances in DNA sequencing provide the opportunity to select and analyse an ample number of different classes of SSRs, in this work, we studied and compared the features and usefulness of EST-SSRs and gSSRs. The data indicated that the two SSR classes reveal different levels of polymorphism in *P. domestica*. EST-SSRs had significantly lower values in the allele-based parameters such as number of alleles and the Simpson’s index of diversity. Nonetheless, the genotypic evenness and the observed heterozygosity were comparable. The latter can be explained by the hexaploidy of the European plum, because a polyploid locus harbours a larger amount of diversity than a diploid one, thus allowing a relatively reduced number of alleles to reveal a high level of heterozygosity in a population. The absence of differences in parameters that strongly depend on allele distribution, such as the evenness and expected heterozygosity, implies that the frequency of the alleles at each locus displays variation that cannot be explained considering the number of alleles. The lack of allele dosage in the molecular screening of polyploids poses limitations in the interpretation of frequency-based analysis (Meirmans et al. 2018), and it is expected to result in an overestimation of the genetic diversity. Nonetheless, the data may also suggest that, in European plum, the occurrence of rare SSR-alleles is not strongly dependant on the class of the marker.

Our study agrees with the literature highlighting the presence of a difference between EST-SSRs and gSSRs when the comparison is carried out within a plant species. gSSRs are generally considered more polymorphic, and this feature has been typically explained in an evolutionary framework related to the natural selection of molecular diversity. However, for a population of clonally selected varieties, it is predicted that clonal propagation will increase the non-random association of alleles at different loci (Birky 1996), making less pertinent the distinction between neutral and non-neutral markers. Our work indicated a large difference in the number of alleles, which is similar, in relative terms, to the one described in chestnut (Martin et al. 2010). In crop plants subjected to intense breeding, such as cereals, cucumber, and sugarcane, the number of gSSR alleles was slightly higher (Xinquan et al. 2005; Hu et al. 2011; Pinto et al. 2006) or similar to the EST-SSRs alleles (Parthiban et al. 2018). In tomato, EST-SSRs had more alleles than gSSRs (Zhou et al. 2015). Overall, it is likely that the amplitude of the difference between the number of EST-SSRs and gSSRs positively correlates with the genetic diversity of the populations/species under investigation, as also suggested in barley (Zhang et al. 2014). For cultivated *P. domestica* it is also necessary to consider that in a clonal population, the alleles at one locus independently accumulate mutations over time (Jarni et al. 2015). Many microsatellites were present in plant genomes long before domestication (Morgante et al. 2002). It is not possible to exclude that a pre-existing difference in the allele number of gSSR and EST-SSRs (dependent on the non-neutral selection of transcribed sequences) in a hypothetical European plum pre-domesticated population has been equally enlarged by asexual propagation, resulting in a large, yet different, number of alleles for both SSR classes. Clonal propagation in polyploids is expected to favour the maintenance of neo‐functionalized and functionally inactive variants of sequences present in multiple copies (Lynch and Conery 2000).

To evaluate the ability to designate relationships between individual, we produced matrices of resemblance to build dendrograms. The data indicated that gSSR have a higher discriminating power and allowed to distinguish all the genotypes under investigation. Nonetheless, the MLG accumulation curve indicated that adding few EST-SSRs can achieve a similar result than gSSRs. In barley, an equivalent rate of discrimination of 12 gSSRs was achieved with 17 EST-SSRs (Leigh et al. 2003). Overall, irrespective of their origin, a handful of SSRs is sufficient for discriminatory purposes in European plum, mostly because of the high number of alleles per locus. The genetic distances showed by the gSSR were on average higher than those revealed by EST-SSRs and this feature associates with the number of diverse alleles of each class. Unlike what is reported in herbaceous plants (Xinquan et al. 2005; Hu et al. 2011; Pinto et al. 2006), in our study, the distances and the cophenetic matrices weakly correlated, implying that the two SSRs classes sampled a different diversity. The fixation of agronomically valuable phenotypes through clonal selection and agamic propagation of the tree allows to preserve adaptive and neutral genetic diversity, but also makes difficult to disentangle the evolutionary selections of DNA variation. Further studies will have to clarify whether the different diversity highlighted by the two SSR classes (at least partially) correlates to different evolutionary forces driving the selection of DNA variation (e.g., natural vs. artificial).

In conclusion, detecting and quantifying differences in polyploid genomes still represent a challenge. In European plum, gSSR and EST-SSRs provide different estimates of some of the genetic parameters calculated at locus level. Larger differences are present with respect to the estimation of the pairwise genetic resemblance. The two classes of SSRs should be considered complementary. gSSRs should be preferred for measures of genetic diversity and for discrimination purposes, considering that highly polymorphic loci provided better estimates of genetic distances (Kalinowski 2002). Nonetheless, the data also indicated that the two SSR classes provided weakly correlated estimates of genetic distance, making useful the contribution of the EST-SSRs not only for facilitating the study of recent adaptation or specific plant traits but also for the effective description and conservation of genetic resources.

## Electronic supplementary material

Below is the link to the electronic supplementary material.

Supplementary file1 (JPG 495 KB)

Supplementary material 2 (PDF 81 kb)
