# Supplementary figures and images for: Comparative analysis of genomic- and EST-SSRs in European plum (Prunus domestica L.): implications for the diversity analysis of polyploids
Source: 3 Biotech. 2020 Nov 21;10(12):543. doi: 10.1007/s13205-020-02513-w (PMC7679426; doi:10.1007/s13205-020-02513-w)

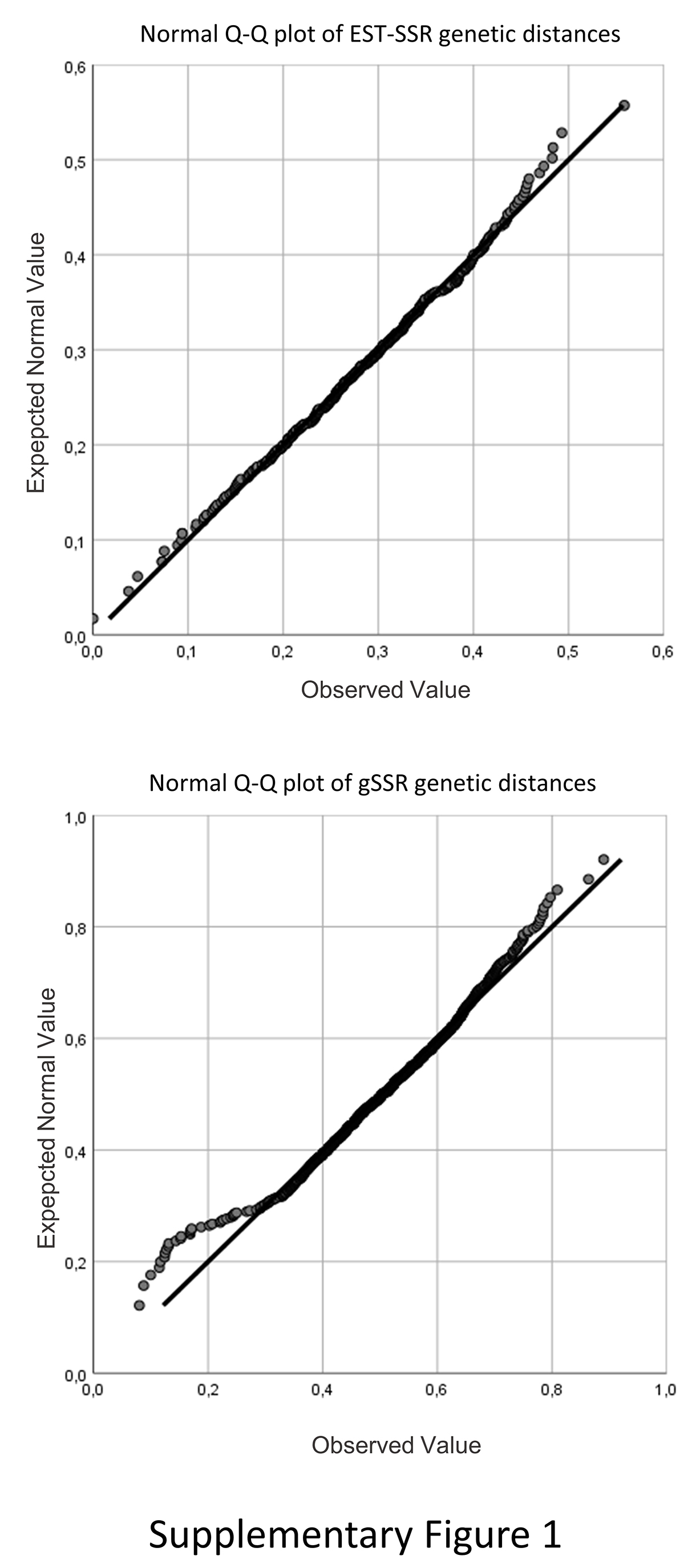

Supplement: Supplementary file 1 — Supplementary file1 (JPG 495 KB) [file 13205_2020_2513_MOESM1_ESM.jpg]
