# Supplementary material for: Comparative analysis of genomic- and EST-SSRs in European plum (Prunus domestica L.): implications for the diversity analysis of polyploids
Source: 3 Biotech. 2020 Nov 21;10(12):543. doi: 10.1007/s13205-020-02513-w (PMC7679426; doi:10.1007/s13205-020-02513-w)
Supplement: Supplementary file 2 — Supplementary material 2 (PDF 81 kb) [file 13205_2020_2513_MOESM2_ESM.pdf]

Supplementary Table 1: main features of the plum varieties under investigation.

Variety	Skin Color [1]	Flesh Color [2]	Fruit Shape [3]
Biancolella di Ottaviano	Y	Y	ELLI
Botta a muro bianca	Y	Y	CIRC
Cacazzara	V	R	OBLA
Calavrice	NA	NA	NA
Coglie ‘e astag bianca	Y	Y	CIRC
Coglie ‘e astag nera	R	R	CIRC
Core	R	O	ELLI
Del Carmine	GY	Y	ELLI
Della Maddalena	NA	NA	NA
Di Spagna	GY	Y	CIRC
Fele	VB	Y	ELLI
Fiaschetta	VB	G	OBOV
Fiocco bianco	Y	Y	ELLI
Fiocco rosa	OR	Y	ELLI
Genova giallo-verde	GY	Y	ELLI
Lecina tonda	Y	Y	CIRC
Marchigiana	R	O	CIRC
‘Mbriaca	GY	GY	ELLI
Melella	R	O	OBLA
Nera tardiva	NA	NA	NA
Occhio di bue	GY	Y	CIRC
Pannaranese	GY	Y	ELLI
Pappacona	Y	GY	ELLI
Pappacona gialla	Y	Y	ELLI
Pappacona rossa	VB	GY	ELLI
Pappacona verde	GY	GY	ELLI
Pazza di Somma	Y	Y	OBOV
Pezza rossa	VB	O	OBLA
Preta ‘e zucchero	R	O	CIRC
Prunarinia	Y	Y	ELLI
Rachele	GY	G	ELLI
Riardo	VB	O	OBOV
San Giovanni	Y	G	OBOV
San Rafele	GY	GY	ELLI
Santa Maria	R	O	CIRC
Santangiolese	NA	NA	NA
Santa Paola	R	O	CIRC
Scarrafona	VB	G	ELLI
Scauratella	Y	Y	CIRC
Sile	GY	Y	ELLI
Turcona	Y	Y	ELLI
Uttaiana	Y	Y	OBOV
Zi' Augusto	GY	GY	CIRC
Zuccarina	Y	Y	ELLI

1 GY: green-yellowish; NA: not available; OR: orange yellow; R: red; V: violet (from light to dark); VB; violet blue; Y: yellow.

2 G: green; GY: green-yellowish; NA: not available; O: orange; R: red; Y: yellow.

3 Circ: Circular; Elli: Elliptic; Obla: Oblate; Oblo: Oblong; Obov: Obovate; NA: not available.

Supplementary Table 2. Primer sequences (in alphabetic order) and their annealing temperature (Ta).

References are provided in the main text.

Locus	Primer forward (5'-3')	Primer reverse (5'-3')	Ta (°C)
BPPCT 004	CTGAGTGATCCATTTGCAGG	AGGGCATCTAGACCTCATTGTT	57
BPPCT 014	TTGTCTGCCTCTCATCTTAACC	CATCGCAGAGAACTGAGAGC	57
BPPCT 028	TCAAGTTAGCTGAGGATCGC	GAGCTTGCCTATGAGAAGACC	57
EPPISF001	TCCCAAGTGGCTACGAGTGT	AGGATGTGGAAGAAGGCAAG	57
EPPISF004	CTGAACCACACAAGCCACAT	TGAGATGAGATGAGAGCAAGC	57
EPPISF027	TTACATGCCTTACGCTGCTG	CACCCAGATGGTCCTGATCT	57
ES4	CCGTAATAACCACCGTCC	CCGCCTTCATCATCCTCT	58.2
ES5	CCAGATCCACTATTTCTTC	GTGTTAGAGCCAGAAACC	56
PS12A02	GCC ACC AAT GGT TCT TCC	AGCACCAGATGCACCTGA	55
UDP98-409	GCTGATGGGTTTTATGGTTTTC	CGGACTCTTATCCTCTATCAACA	57
